# Ethnoherpetological notes regarding the paha frogs and conservation implication in Manaslu Conservation Area, Gorkha District, Nepal

**DOI:** 10.1186/s13002-019-0304-5

**Published:** 2019-05-10

**Authors:** Biraj Shrestha, Min Bahadur Gurung

**Affiliations:** 1 Resources Himalaya Foundation, Naya bato, Sanepa, Ring road, Lalitpur, Nepal; 2 Small Mammals Conservation and Research Foundation, Kumaripati, Lalitpur, Nepal

**Keywords:** Conservation, Ethnoherpetology, Manaslu, Paha, Frog, Nepal

## Abstract

**Background:**

Paha hunting is a commonplace recreational activity in the mountainous regions of Nepal. The collection is primarily for food use and secondarily preferred as medicinal forms, and utilized by many ethnic groups: Magar, Rai, Gurung, Jirel, etc. in different parts of the country. In this study, we documented the ethnoherpetological relationship of the local community with paha frogs in Manaslu Conservation Area, Gorkha District, Nepal. We confirmed the use of three species of paha, namely *Nanorana liebigii*, known locally as Man paha, *Ombrana sikimensis*, and *Amolops formosus* by the local community and recorded information on paha hunting strategy, meat preparation and storage techniques, zootherapeutic benefits, quantities harvested, and population status perception.

**Methods:**

We conducted our fieldwork in the period between April 2016 and March 2017 in major settlements of Sirdibas, Chumchet, Bihi, and Prok villages. We interviewed 50 people (39 males and 11 females) using a semi-structured questionnaire format and recorded open interviews with potential informants. Our survey focused mainly over Sirdibas village inhabited by Gurungs.

**Results:**

People usually hunted paha in Spring (March to May) and Summer (June to August) season either by flashing torchlight at night time (45.7%) or flipping big rocks under the water (29.6%). *Nanorana liebigii* (50%) is highly preferred for its dual purpose of delicacy and medicine, while *Ombrana sikimensis* (33.33%) solely considered for food and *Amolops formosus* (16.67%) for medicine. Majority of the people (43.90%) collected 51–100 individuals of paha at one hunting season and sold locally in the price range between NPR 50–250 (USD 0.45–2.26). People opined paha numbers have diminished over the last decade (76%), suggested strict regulation of hunting (58.5%), and educational campaigns (29.2%) as measures of protection.

**Conclusion:**

Our results demonstrated the difference in ethnoherpetological relationship among the Gurung community in lower Sirdibas village and the Tibetan Lama community in Manaslu. Since frogs around the world are in rapid decline, it is imperative that recreational killings of paha need to be checked with regulatory mechanisms across Nepal. There is an urgent need to shelter paha frogs under wildlife protection regulation and prioritize for conservation.

## Background

Humans and herpetofauna (amphibians and reptiles group) interaction have existed globally for millennia, making it an important discipline of ethnozoological study. The level of interpretation is often influenced by the physical environment, cultural norms, and personal experience [1]. Ethnoherpetology is a sub-branch of ethnozoology where human relationship with amphibians and reptiles are studied. Such studies are crucial in evaluating human impacts over the exploited species so as to inform conservation management plans and have become a grown interest among the resource managers, planners, and decision-makers [2, 3]. The rising human populations and growing demands have rendered enormous pressure over resources exploitation and have ultimately threatened some wild species with extinction, including frogs [1]. In many rural societies, frogs are collected from the wild and consumed as a source of protein, while others prefer it as a matter of delicacy [4]. Thus, the analysis of ethnozoological information is becoming more important when traditional medicine is the primary health treatment facility for over 80% of the world’s population, and the bush meat contributes around 80% of the total meat consumption in rural communities [5, 6].

“Paha” is a generic term used in the hilly regions of Nepal for stream-dwelling frogs represented by the genus *Nanorana* (former *Paa*), *Ombrana*, and *Amolops* and harvested by the local community [7]. The frogs inhabit shallow mountain streams with pool and riffle attributes to torrential cascades, often sheltering beneath the rocky bottoms and crevices during the day time. Paha frogs are collected basically for food and therapeutic purposes, and often, paha hunting is seen as a form of recreational activity [8, 9]. However, there has been relatively little attempt to document such forms of human-frogs’ interaction in Nepal.

Although few ethnozoological studies in Nepal mentioned the use of herpetofauna, detailed ethnoherpetological documentation is rare [10–13]. Hunting paha for delicacy is seen as a grave threat for the survival of frogs in the mid-hills of Nepal, as there are no regulatory mechanisms to control such overharvesting [7]. Despite tremendous collection, there is no information on the statistics of such harvest, trade, and the associated impacts. Additionally, amphibians around the world are in serious danger due to individual or synergistic effects of key drivers such as habitat loss, pollution, infectious disease, invasive species, climate change, dissection purpose, etc., and currently, one-third of the global amphibian species (7994) are facing higher risks of extinction [14]. Thus, the general goal of this descriptive study is to aid in paha conservation of Nepal through an attempt to document (1) the ethnoherpetological relationship of the local community with paha frogs, (2) contemporary status of paha hunting, and (3) conservation implications in Manaslu.

## Methods

### Study site

**Fig. 1:**
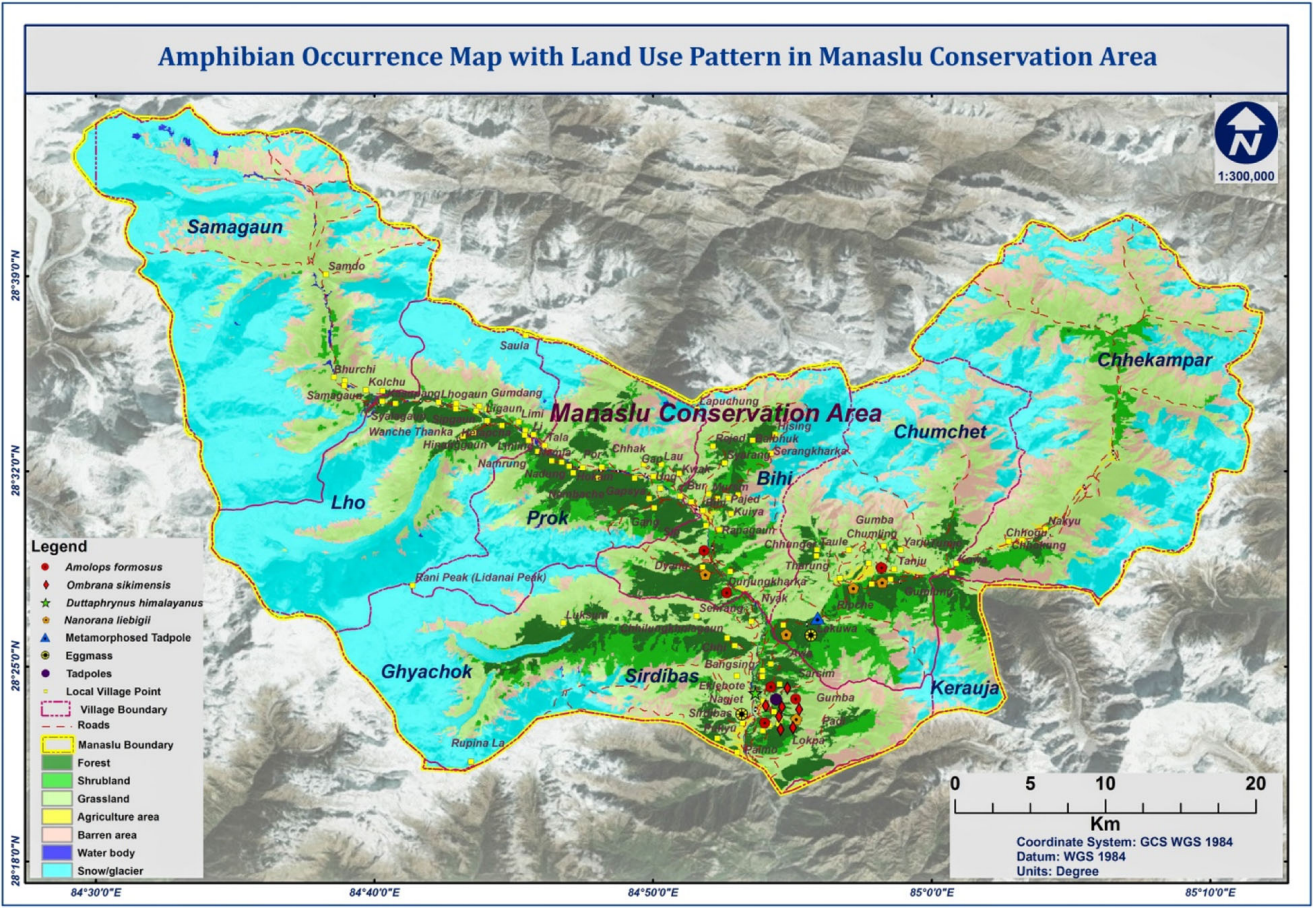
Geographical location of the study sites: Sirdibas, Chumchet, Bihi and Prok (northern Gorkha district)

Manaslu Conservation Area (MCA) is a high land protected area that lies in the northern part of Gorkha district and spread over an area of 1663 km^2^ in the Mansiri range of the Himalayas [15]. MCA starts with an altitude as low of 1400 m above sea level (asl) to the summit of Manaslu, 8163 m asl and has given rise to five different bio-climatic zones allowing 19 types of ecosystems and 11 types of vegetation that harbors rich taxa of plants and animals [16]. The region is expected to shelter 22 species of amphibian and reptile, of which four species of amphibian are locally called paha [8]. We collected ethnoherpetological information regarding paha from settlements in major villages: Sirdibas, Chumchet, Bihi, and Prok regarded formerly as the Village Development Committees (Fig. 1). These sites were selected after confirmation of paha occurrence following our amphibian survey and also reported by the local people.

### Demographic profile

Sirdibas is a thickly populated settlement with 2510 people living in total 572 households [17]. Gurungs are the major ethnic groups in Sirdibas having their own dialect, unlike Gurungs in other parts of the country [18]. They practice both Hinduism and Buddhism religions. While, Chumchet (266 households; 928 total population), Bihi (208 households; 612 total population), and Prok (187 households; 575 total population) villages are inhabited by specific ethnic groups (Tsumpa, Kutangpa, and Nubripa) based on the name of the valleys: Tsum in the east, Kutang, and Nubri in the western side. Their origins are traced to be in Tibet having distinct Tibeto-Burman dialects and follow Tibetan-Buddhism [18–20]. The local community in MCA is highly marginalized due to geographical remoteness of the area that cuts off access to basic amenities like electricity, drinking water supply, good health facilities, transportation, and educational institutions. Majority of the people depend upon subsistence agriculture, livestock keeping, and natural resources with no other means of economy [21]. However, the region saw a rise in hotels after significant improvements in trail infrastructure to promote tourism in the area, but the access to benefits is limited largely to outsiders who run hotel businesses and trekking companies.

### Data collection

Our survey in MCA took place between April 2016 and March 2017. We collected information regarding ethnozoological use of paha (food and medicine), cultural beliefs, underlying threats, hunting season, quantities of collection, and measures of protection. For the purpose, we used a semi-structured questionnaire format including dichotomous questions and conducted both snow ball sampling [22] and open interviews [23].

The questionnaire interview was focused mainly on Sirdibas with Gurung community based on the local available information that paha hunting occurs mostly in this region of Manaslu. The interviewees varied in their ages from 13 to 60 years old. We selected respondents regarding their experience with paha hunting, local use, and traditional medicinal knowledge. In total, we interviewed 50 respondents (39 males and 11 females) from MCA including youth, school children, elder people, MCA staffs, and religious leaders (Table 1). We had also taken photographs of the paha species hunted in the region and recorded other insightful information.

**Table 1:** General profile of the respondents (*N* = 50)

Category		Frequency	Percentage
Gender			
Male		39	78
Female		11	22
Age group			
≤ 20 years		8	16
21 to 40 years		17	34
41 to 60 years		25	50
Education			
Primary (grade 1–5)		15	30
Lower secondary (grade 6–8)		7	14
Secondary (grade 9–10)		8	16
Higher secondary (grade 11–12)		4	8
University (Bachelors and Masters)		3	6
No response		13	26
Occupation			
Agriculture		19	38
Business		11	22
Service		9	18
Student		8	16
None		3	6
Ethnicity			
Gurung		42	84
Tibetan Lama		6	12
Dalit		1	2
Chhetri		1	2

More than 50% of the respondents had little access to education due to lack of schools and availability of only one secondary school in the whole MCA. A large number of respondents are from the Gurung ethnicity (*n* = 42, 84%). On the other hand, Tibetan Lamas from the monasteries have designated surrounding higher regions as kill-free zones and prohibited killing any kind of animals in the region. Their response, however, has been included to garner cultural beliefs regarding paha from the region.

Since the collected information was mostly qualitative in nature, we then coded the data, analyzed it using SPSS 16.0, and computed the frequencies for a group of cases and also for separate variables in the case of multiple responses.

## Results and discussion

**Fig. 2:**
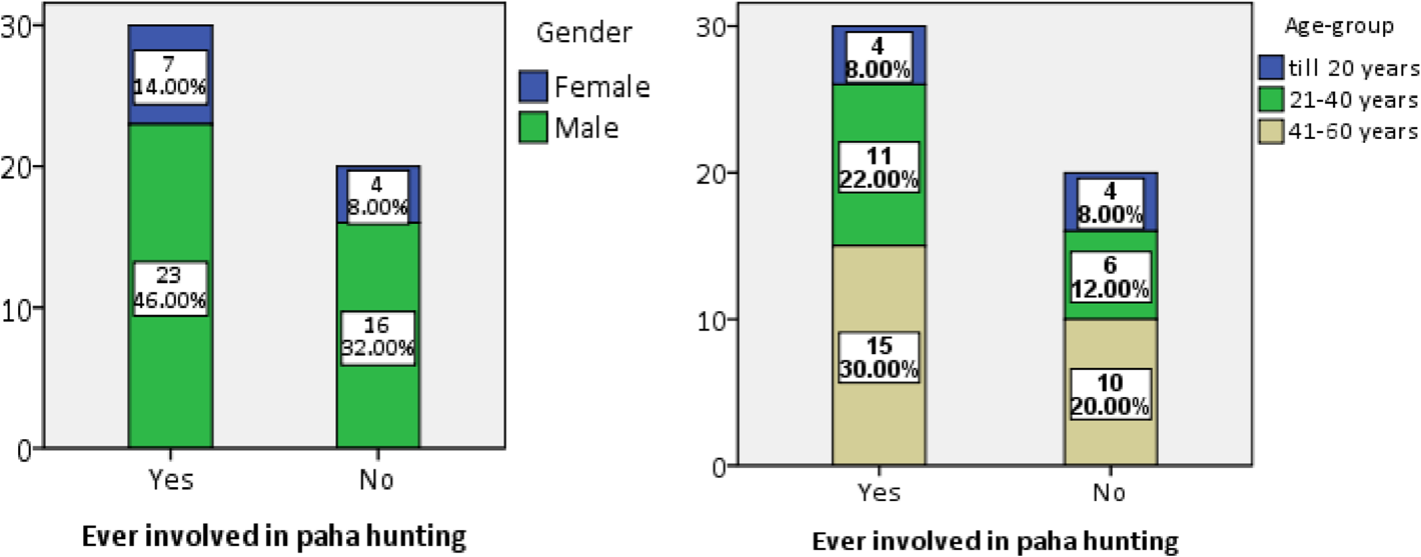
Respondents count and percentages involvement in paha hunting segregated by gender and age group

Sixty percent of informants have gained experience in paha hunting with greater percentage of males, i.e., 46% (Fig. 2). Like fishing, hunting paha is dominated by male members of the community than females in Manaslu, and ethnozoological studies have confirmed that hunting/fishing is largely restricted to men assimilating a good deal of knowledge about the harvested species, their general biology, and ecology [24]. The age-group distribution among the hunters showed that people between 41 and 60 years old have greater experience (30%), followed by people in the range of 21–40 years old (Fig. 2). These two age-groups hold rich ethnoherpetological knowledge regarding paha frogs, irrespective of the purpose of hunting. However, the aged people in rural communities around the world are found to have greater experience with zootherapeutic knowledge of animals than younger generations due to modernization, absence of knowledge handover, and failure to preserve traditional ecological knowledge [25].

People usually hunt during the Spring (March to May) and Summer/Monsoon (June to August) season which coincides with the breeding and metamorphosis stage of the paha. At this time of the year, paha are observed near the water bodies and calling out at nights. Few people even attempt searching paha in winter; however, it requires ample physical effort as it is their hibernation time.

Local people in Sirdibas hunt paha using different strategies and depend upon seasonal calender. People flash lights over the paha frog resting somewhere nearby streams at night time, followed by handpicking it. This technique (45.7%) is highly favored over others like rock flipping (29.6%) and damming (17.3%) that demands physical effort (Table 2). Flipping and damming are used when the water flow remains low (in Spring and Winter) and mostly during the day time. Some people also use multi-pronged bamboo spear (similarly used for fishing) to kill paha that are either underwater or clung at the edge of the precipice in mountain streams.

**Table 2:** Percentage share of response for paha hunting methods

SN	Paha hunting techniques	Responses	
		*N*	Percentage (%)
1	Torching	37	45.7
2	Flipping big rocks	24	29.6
3	Damming streams	14	17.3
4	Bamboo spears	6	7.4
Total		81	100.0

### Ethnoherpetological notes

**Fig. 3:**
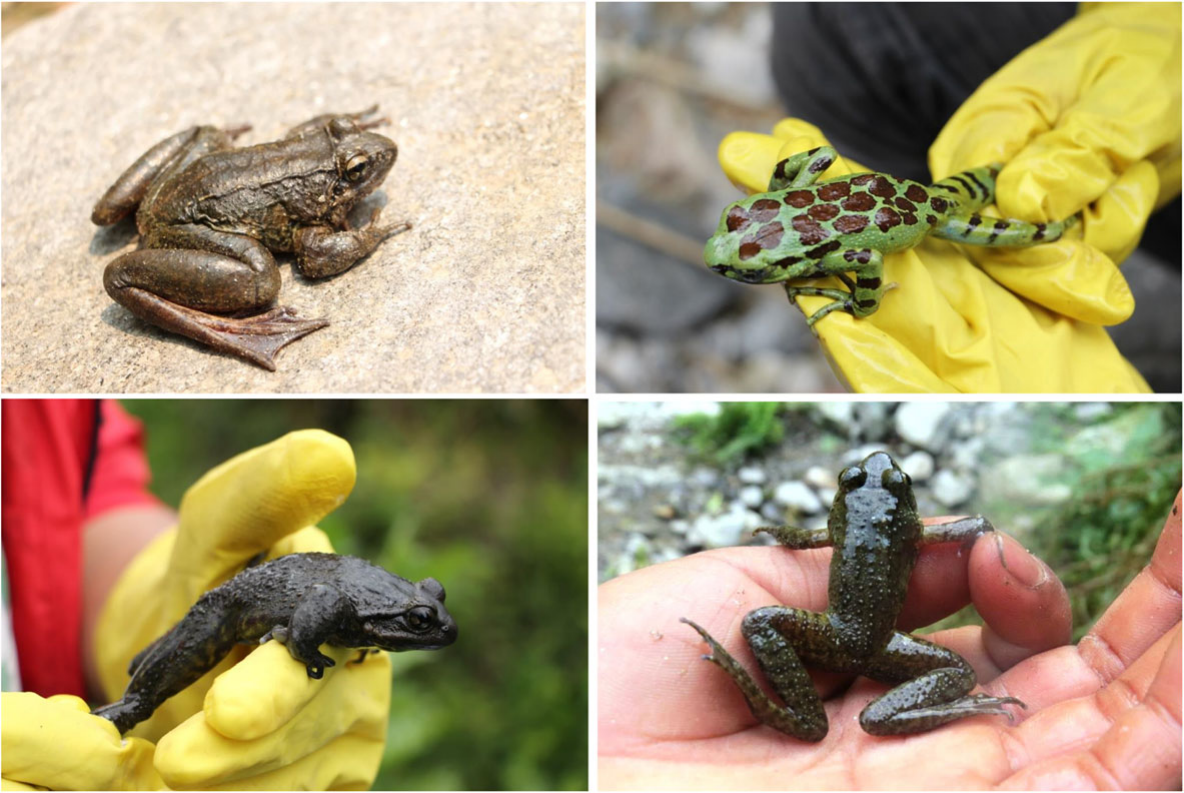
Paha frogs hunted in MCA (clock wise from bottom left); *Nanorana liebigii*, *Ombrana sikimensis*, *Amolops formosus*, and unidentified paha

We confirmed three species of paha, namely *Nanorana liebgii*, *Ombrana sikimensis*, and *Amolops formosus* from two families: Dicroglossidae and Ranidae that have ethnozoological values (Fig. 3). These are the mountainous stream habitat dwelling sympatric frogs that have nocturnal mode of living in river and riparian systems near human settlements. They are largely restricted to their habitats, lay eggs in cluster under water, and complete therein, its metamorphosis.

**Fig. 4:**
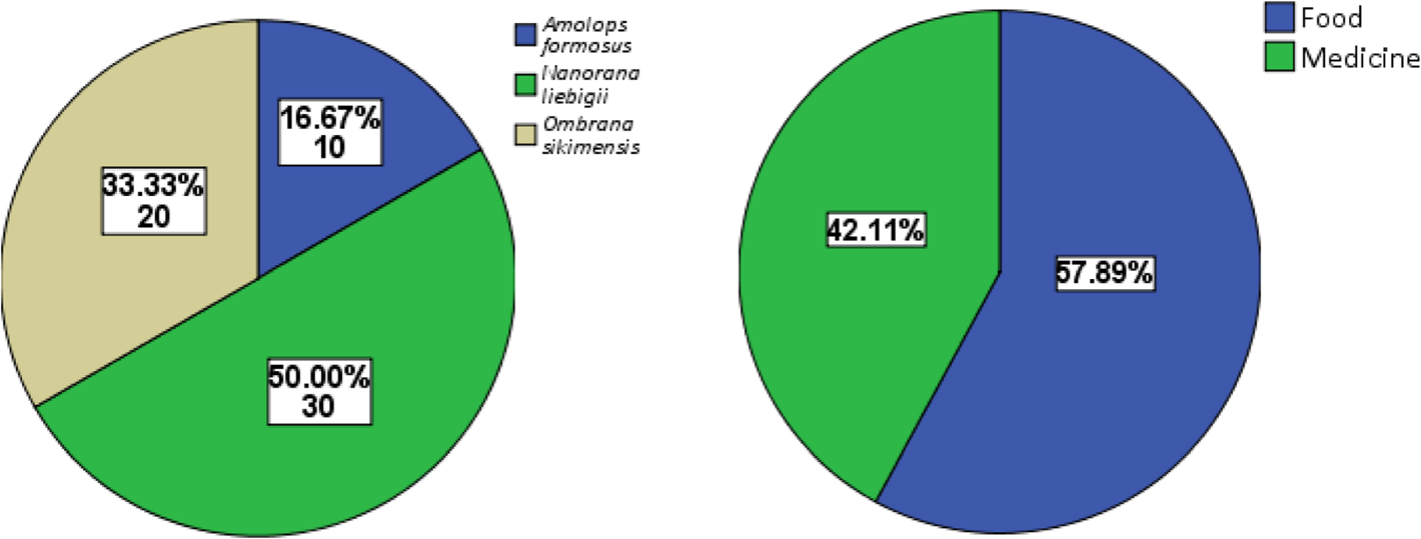
Percentage share of response for species preference and purpose of use

Half of the total responses were in favor to *Nanorana liebigii,* while less than a quarter response was received for *Ombrana sikimensis*. This is explained by preference of single species for different utilitarian values such as food and medicine. Respondents favored food value of paha over the medicinal purpose (Fig. 4). The meat regarded as nutritious, of supreme taste, and available free of cost have ranked its food value high, whereas the reliance over modern medicines coupled with eroding zootherapeutic knowledge has diminished traditional medicinal knowledge of all kind.

**Fig. 5:**
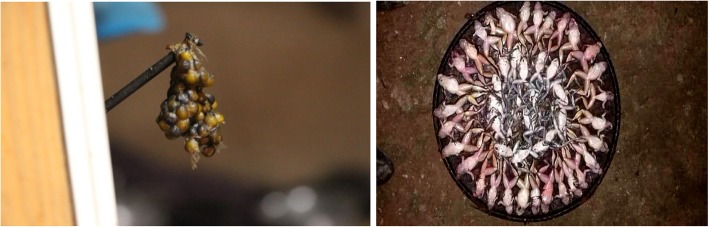
(Left) Dried eggs of Man paha; (Right) Freshly killed paha laid in clusters

Table 3 outlines the food and medicinal usage of paha frogs found in MCA along with their vernacular names, body parts used, preparation techniques, therapeutic benefits, and the IUCN status. *Nanorana liebigii*, popularly known as “Man paha” serves both food and medicinal purposes, thus the hunting pressure over it has been excessive. There are earlier reports of paha usage as food and medicine in Manaslu, especially Man paha, while *Ombrana sikimensis* for food and *Amolops formosus* having healing properties [8, 26, 27]. Harvesting paha for its usefulness as food and traditional medicine by different ethnic groups have been discussed in a collection of literatures across Nepal [9–13, 26]. From the study, paha used for food and medicines are consumed in a variety of ways. After removing guts, the whole body is deep fried using oil and mixed with various types of spices. It can either be smoked above fire or left to dry out in the sun/shade for future use. However, people preferred consuming cooked fresh meat and smoked paha as the best way (Table 4). Respondents opined that consuming the meat supplements the body with a good surge of energy especially for sick people, pregnant women, and nursing mothers. Dried eggs of the Man paha is preserved in shade and consumed afterwards in the belief that it helps to regain sexual power (Fig. 5). Man paha's meat is referred to as a panacea for curing typhoid, diarrhea, dysentery, and a number of different ailments. However, respondents did not have a preference for *Amolops formosus* as food due to its strong bitter taste resulting from the pungent slime off its body. The slime, however, is used by local people for healing open cuts and recovering wounds.

**Table 3:** Ethnozoological use of paha among local community in Manaslu Conservation Area, Gorkha District

Scientific name	Common name	Nepali name	Vernacular name	Use (F, M)^a^	Parts used	Procedure	Therapeutic purpose	IUCN Red List status
*Nanorana liebigii* (Günther, 1860)	Liebig’s paa frog	Man paha	*Luklang*, *Myakluk*	F, M	Eggs and all body parts: flesh, legs, bones, skin, etc. except guts (intestine)	Fresh raw meat deep fried in hot oil and mixed with spice for curry, either smoked or sun/shade dried (including eggs) for later use.	Meat consumption supplies strength and promotes vigor for pregnant women, nursing mothers and individuals recovering from illness. Treats typhoid, diarrhea, dysentery, stomach ache, headache, fever, cough-cold, urine problem, asthma, etc. Skin used as antiseptic for healing wounds and crushed paha bones for treating fractures. Dried eggs cure impotency.	Least Concern (LC), Trend Decreasing
*Ombrana sikimensis* (Jerdon, 1870)	Sikkim Asian frog	Rato paha		F	All body parts: flesh, legs, bones, skin, etc. except guts (intestine)	Fresh raw meat deep fried in hot oil and mixed with spice for curry, either smoked or sun/shade dried for later use		Least Concern (LC), Trend Decreasing
*Amolops formosus*^b^ (Günther, 1876 “1875”)	Assam cascade frog	Hariyo paha	*Raslang*	M	Skin, slime, and eggs	Freshly collected skin secretions and peeled skin	Skin and fresh eggs used as antiseptic for healing wounds.	Least Concern (LC), Trend Decreasing

^a^Represents food and medicinal

^b^Avoided for consumption due to strong odor and bitter taste

**Table 4:** Percentage share of response for local techniques of meat processing

SN	Meat processing method	Responses	
		*N*	Percentage (%)
1	Sun dry	9	19.6
2	Smoked	18	39.1
3	Fresh meat	18	39.1
4	Peeling the skin	1	2.2
Total		46	100.0

### Current status

**Fig. 6:**
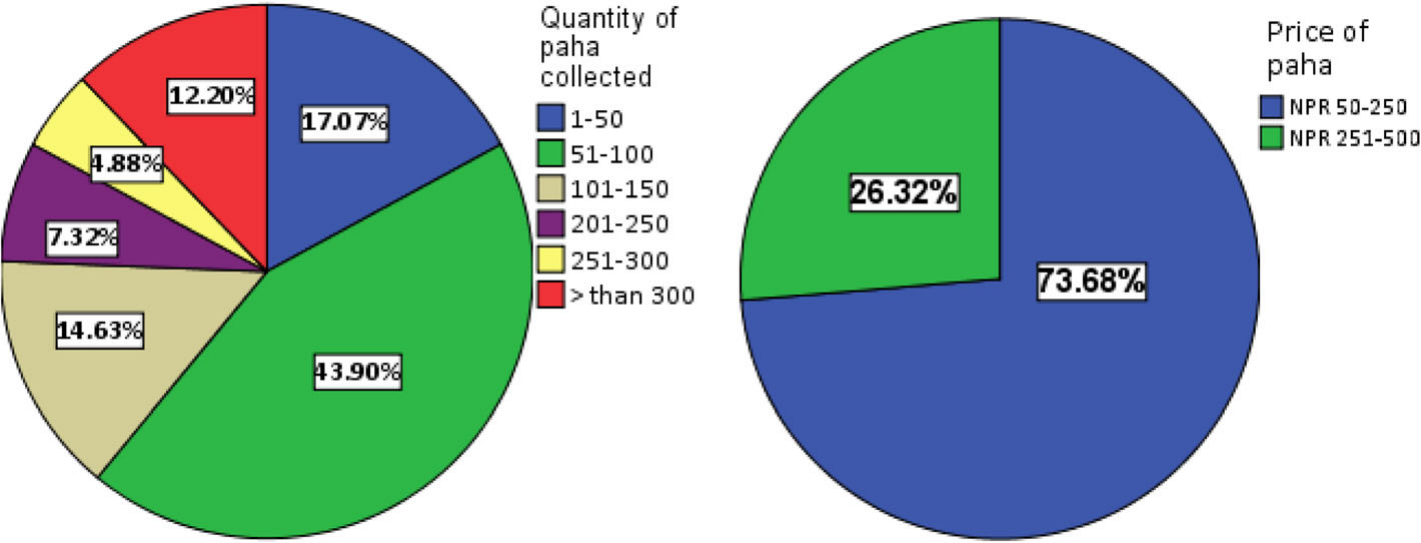
Percentage share of response for quantity of paha collected and price per individual

In this study, majority of the people (43.90%) generally collected paha between the ranges of 51–100 individuals at a single season (Fig. 6). These collections are purposively meant for recreational food use rather than for protein requirements or for zootherapy. This is because the meat from chicken and livestock raised by the local community are the source of protein in Manaslu while Sirdibas has a well-running health post for medical facility. The collected frogs are traded locally for a price between NPR 50–250 (USD^1^ 0.45–2.26) for one individual (Fig. 6). Sometimes, when kids collect paha from the streams, adults persuade them to exchange the frogs for a packet of noodle.

### Conservation implication

**Fig. 7:**
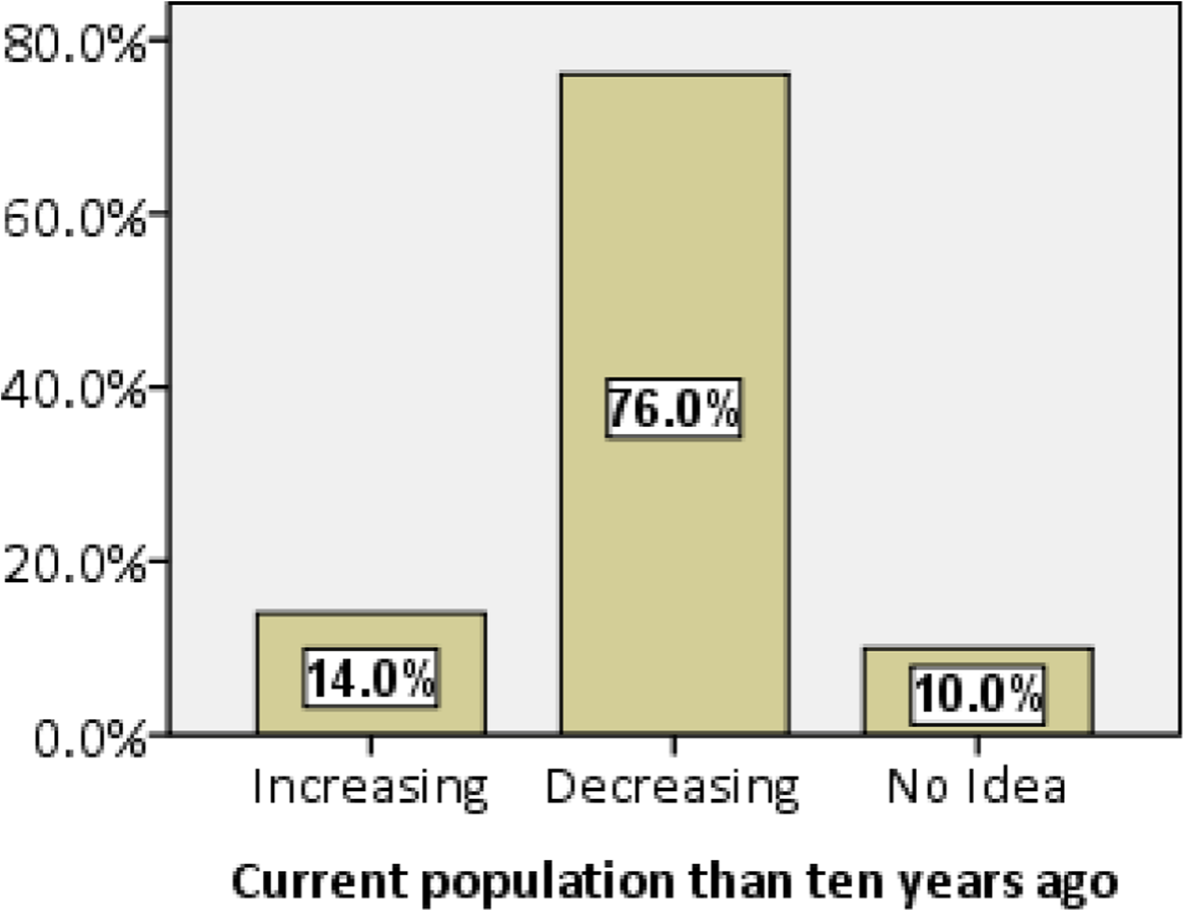
Percentage share of opinion on the status of paha population in MCA

Although the IUCN Red List Authority has assessed all the three species of paha frogs in the Least Concern (LC) category, their population trend is decreasing (Table 3). This is corroborated with our findings where over 75% of the respondents claim that paha population is decreasing in Manaslu (Fig. 7). On further inquiry, informants stated unsustainable hunting to be the leading cause of decline (44%) and followed by streams drying up (34.1%) in the MCA through diversion of water for communal use, hotel business, and agriculture (Table 5).

**Table 5:** Percentage share of response for reasons of paha decline in MCA

SN	Drivers of paha decline	Responses	
		*N*	Percentage (%)
1	Unsustainable hunting	40	44
2	Landslide	18	19.8
3	|Streams drying up	31	34.1
4	Pollution	2	2.18
Total		91	100.0

Frogs are rapidly disappearing animals on this planet with nearly 32.5% of the global amphibians threatened with extinction, and this figure will keep continuing to rise in the future [28]. In Nepal, overcollection is one of the major threats to frogs' decline among other drivers such as land use change, pollution, pesticides use, dissection purpose, etc. [7]. Wildlife hunting for utilitarian purpose is largely an anthropogenic pressure that affects the natural population of the target species and bears ecological implications mainly by disrupting food chain [3]. Frogs act as both a predator and a prey to a number of animals, thus maintaining healthy ecosystems. As biological pest controllers, frogs check the population of agricultural pests and disease-carrying vectors such as mosquitoes and ticks. In the absence of frogs, these pests might become overabundant [29]. Further, frogs provide valuable ecosystems services in aquatic habitats through alterations in primary productivity, nutrient cycling and deposition, and cleaning waterways [30]. Having moist skin, frogs are considered as bio-indicators of the environmental quality and thus, their presence is an indication of the healthy riparian habitats [31].

There are neither any specific regulations that shelter frogs for protection nor any collaborative amphibian themed educational intervention in Nepal, which led to exploitation to this group of animals at a greater extent. However, Nepal is obligated to provide some forms of protection to amphibians as being a member country to the Convention of International Trade in Endangered Species of Wild Fauna and Flora (CITES) and regulations such as Aquatic Animals Protection Act 1961, National Parks and Wildlife Conservation Act 1973, and Environmental Protection Act 1996 that accommodate biodiversity in general [7]. In Manaslu, religious taboo in higher regions has helped to protect all forms of animals including paha frogs. The Lama leader from the monastery has declared higher regions of Manaslu as kill-free zones in all three valleys: Nubri, Kutang, and Tsum. Buddhists venerate paha as the daughter of the sky god and in case paha is killed, it is believed that the god will be upset and the villagers shall face a bad omen. This taboo has motivated the local youth club in Lokpa, lower Tsum valley in Chumchet to punish anyone involved in paha hunting either through a fine of NPR 50,000 (USD 452.37) per person or spend one to two nights in a community detention center at Lokpa.

In Sirdibas, respondents felt that paha must be conserved before it is too late owing to decreasing population. Over 55% of the informants opined that the unsustainable hunting must be checked, while 29.2% of the respondents realized the need for amphibian awareness campaigns (Table 6). Despite MCA is a protected area, conservation concern for paha frogs are found very minimal to zero action by the Manaslu Conservation Area Project (MCAP) staffs, in the case of paha hunting.

**Table 6:** Percentage share of response for paha conservation means

SN	Measures of protection	Responses	
		*N*	Percentage (%)
1	Regulate hunting	38	58.5
2	Awareness campaigns	19	29.2
3	Conservation of aquatic sources	8	12.3
Total		65	100.0

## Conclusion

Paha frogs are important entities of ethnozoological studies as they have well-established ethnoherpetological connection with rural communities, as is the case of Manaslu. Our study documented the use of paha for food as a delicacy and its meat as a cure for minor ailments to the Gurung community, while the same animal is revered sacred by Tibetan Lama community. This sets an example of how different cultural norms in adjacent communities have contrasting views on the same group of animals. Such pieces of information are critical for the conservation of paha frogs from this region. However, additional studies are necessary regarding the economic chain of paha trade, identifying the demand and supply, and the end users to gain a broader picture of such wildlife based markets.

Since the use of paha for food and medicines may have substantial harvesting implications to the wild stocks, there must be a concerted effort from stakeholders of the region to control overexploitation of the affected species. The cases of paha hunting and use must be addressed seriously where ever possible all across Nepal, and paha conservation message should be embedded in environmental education programs that aspire to change people’s attitude towards conservation and sustainable use of biological resources. High priority must be given to species that are exploited widely by human societies.

## Footnotes

1 USD 1 = NPR 110.53
